# Influence of Intensified Supervision by Health Care Inspectorates on Online Patient Ratings of Hospitals: A Multilevel Study of More Than 43,000 Online Ratings

**DOI:** 10.2196/jmir.5884

**Published:** 2016-07-15

**Authors:** Rudolf Bertijn Kool, Sophia Martine Kleefstra, Ine Borghans, Femke Atsma, Tom H van de Belt

**Affiliations:** ^1^Radboud University Medical CenterRadboud Institute for Health Sciences, IQ HealthcareNijmegenNetherlands; ^2^Dutch Health Care Inspectorate (IGZ)UtrechtNetherlands; ^3^Academic Medical CenterAmsterdamNetherlands; ^4^Radboud University Medical CenterREshape Innovation CenterNijmegenNetherlands

**Keywords:** rating sites, supervision, social media, online reviews, hospitals

## Abstract

**Background:**

In the Netherlands, hospitals with quality or safety issues are put under intensified supervision by the Dutch Health Care Inspectorate, which involves frequent announced and unannounced site visits and other measures. Patient rating sites are an upcoming phenomenon in health care. Patient reviews might be influenced by perceived quality including the media coverage of health care providers when the health care inspectorate imposes intensified supervision, but no data are available to show how these are related.

**Objective:**

The aim of this study was to investigate whether and how being under intensified supervision of the health care inspectorate influences online patient ratings of hospitals.

**Methods:**

We performed a longitudinal study using data from the patient rating site Zorgkaart Nederland, from January 1, 2010 to December 31, 2015. We compared data of 7 hospitals under intensified supervision with a control group of 28 hospitals. The dataset contained 43,856 ratings. We performed a multilevel logistic regression analysis to account for clustering of ratings within hospitals. Fixed effects in our analysis were hospital type, time, and the period of intensified supervision. Random effect was the hospital. The outcome variable was the dichotomized rating score.

**Results:**

The period of intensified supervision was associated with a low rating score for the hospitals compared with control group hospitals; both 1 year before intensified supervision (odds ratio, OR, 1.67, 95% CI 1.06-2.63) and 1 year after (OR 1.79, 95% CI 1.14-2.81) the differences are significant. For all periods, the odds on a low rating score for hospitals under intensified supervision are higher than for the control group hospitals, corrected for time. Time is also associated with low rating scores, with decreasing ORs over time since 2010.

**Conclusions:**

Hospitals that are confronted with intensified supervision by the health care inspectorate have lower ratings on patient rating sites. The scores are independent of the period: before, during, or just after the intervention by the health care inspectorate. Health care inspectorates might learn from these results because they indicate that the inspectorate identifies the same hospitals as “at risk” as the patients rate as underperformers.

## Introduction

Patient rating sites (PRSs) are an upcoming phenomenon in health care [1]. In many countries, websites such as RateMDs, Vitals, and Zocdoc in the United States, Jameda in Germany, and NHS Choices in the United Kingdom have created a platform for patients to share their experiences with health care providers. The number of ratings is growing and it comprehends all kinds of care. Nevertheless, the usefulness of PRSs for health care is being discussed [2-5]. Recently, a scoping review showed a growing body of literature on positive relationships between ratings on PRSs and indicators of quality of care such as patient satisfaction, mortality, and readmissions [6,7]. The content of PRSs is used for several purposes including supervision by the Dutch Health Care Inspectorate (Inspectie voor de Gezondheidszorg or IGZ) since 2015 [8].

Patient reviews are influenced not only by the type of care received by the patient and the way health care is delivered, but probably also by external stimuli such as media coverage. Research has shown that patients, especially the local ones, are influenced in their trust in health care providers by media attention [9]. In the Netherlands, media generally pay close attention to health care providers where “something might be wrong.” Recent examples are 2 hospitals that came under intensified supervision of the IGZ after it noted patient safety problems [10,11]. Intensified supervision means frequent announced and unannounced site visits and consultation with the board, and it will be ended when structural improvements of the quality and safety of care have been proven and the board shows to be in control, see Textbox 1 [12]. In particular, health care magazines and local newspapers and sometimes local or even national radio and television report the intensified supervision and highlight it with special coverage. This could influence the opinion of patients of the health care providers and so their ratings on PRSs.

The fact that a health care inspectorate uses patient reviews as one of the components in risk detection, and that at the same time patient reviews could be influenced by intervention by the health care inspectorate, raises the question of how patient ratings of health care providers change after publication of intensified supervision by the inspectorate. There is no existing research on the extent to which patient ratings of health care providers are affected by intensified supervision of a health care inspectorate. One might expect that mean patient ratings of health care providers under intensified supervision are generally low compared with other health care providers before intensified supervision, because of the quality problems that are found. Patients and their relatives may use low ratings to draw attention to the problems they have experienced. Our first hypothesis was therefore that health care providers under intensified supervision would have a relative low mean overall patient rating in the period before intensified supervision compared with comparable providers.

Second, the mean rating of health care providers under intensified supervision might probably increase during the intensified supervision compared with the period before intensified supervision. Although negative media attention can ruin a reputation, previous research has also shown that the trust of patients in their doctors is high [13] and trust influences loyalty [14]. Patients might support their health care provider by sharing positive experiences and higher ratings. Third, we assumed that after the intensified supervision, when most of the publicity is gone, mean ratings of health care providers would be stable and comparable with others.

To investigate the aforementioned assumptions, we performed a multilevel study with the following research question: To which extent is intensified supervision associated with online hospital patient ratings? Because of the limited number of ratings of other health care providers, we focused on only hospitals in this study.

**The Dutch Health Care Inspectorate.**

The Dutch Health Care Inspectorate (Inspectie voor de Gezondheidszorg or IGZ) is an agency under the Ministry of Health, Welfare and Sport. It is the official regulatory body charged with supervising the quality and safety of health care services, prevention activities, and medical products in the Netherlands. The IGZ has organized its supervision in several ways to ensure compliance with (professional) standards and guidelines and to ensure patient safety. The two most important methods are incident-based supervision and analyses of various types of risk information, also known as risk-based supervision.

The IGZ can also impose intensified supervision on a provider of care, entirely or one of its departments, if the reports from the inspectors, any reports and analyses of calamities, and/or the risk indicators show high risks for quality and/or safety of care and when there is insufficient faith in the strength and effectiveness of the board to realize improvements on time. Intensified supervision includes frequent announced and unannounced site visits and consultation with the board. Intensified supervision will be ended when structural improvements of the quality and safety of care have been proven and the board shows to be in control. When deciding upon the most appropriate enforcement measure, the inspectorate will take the following variables into account:

- the 5 D's: dissatisfaction, discomfort, disease, disability, and death (internationally recognized criteria);
- the number of people at risk (ie, a large, medium, or small risk group);
- the manner in which care provision is organized and structured with a view to quality and safety outcomes (poor, moderate, good); and
- the attitude of the care provider (ignorance, incompetence, noncompliance).

## Methods

### Study Design

We performed an observational study using publicly available data.

### Data Sources

First, we scanned the website of the IGZ where it publicly announces the providers that will be monitored by intensified supervision in order to arrange a list of all intensified supervision hospitals. We collected the names of these hospitals from January 2010 to December 2015, including the exact start and end date of the intensified supervision. In this period the IGZ decided to monitor 7 entire hospitals intensively by intensified supervision and to end it. We excluded 2 hospitals in which only 1 department was monitored by the inspectorate.

Second, we used data from the biggest PRS in the Netherlands, Zorgkaart Nederland, with more than 272,000 ratings and more than a million visitors per month in 2015. We used the publicly available data from January 1, 2010 to December 31, 2015, in which period the IGZ did not yet use hospital ratings to decide on intensified supervision. On Zorgkaart Nederland, patients can rate health care providers on 6 aspects, namely, accommodation, appointments, treatment, information, listening, and professionals, based on a scale from 1-10, where “1” stands for “extremely poor” and “10” for “extremely good.” An overall score is automatically calculated (rounded to the nearest 0.1 percentage point). We used the number of ratings, the percentage of reviews with a score lower than 6.5, and the mean rating score on Zorgkaart Nederland given by patients. The 6.5 threshold is based on the theory of the Net Promoter Score [15]. This theory considers the scores 9 and 10 as positive “promoters,” the scores 7 and 8 as neutral, passively satisfied, and the scores 0 to 6 ratings as “detractors,” or negative recommendations. Because we were primarily interested in the “detractors,” as these ratings might tell something about patient safety, we dichotomized the dependent variable rating scores: scores higher than and equal to 6.5 were labeled “0” and scores lower than 6.5 were labeled “1.”

### Analysis

We compared the data of the 7 intensified supervision hospitals with a control group of 28 hospitals. In the Netherlands, 3 types of hospitals exist: relatively small general acute care hospitals, the bigger teaching hospitals, and the major academic hospitals. The control group hospitals were purposively sampled in the same region (North, South, East, and West) and same type as the intensified supervision hospitals and also on having the most ratings, thus guaranteeing a sufficient number of ratings. The total number of hospitals included in the dataset was 35 with 43,856 ratings. We analyzed the data for the different categories of hospitals because we could expect a difference in rating scores; patient satisfaction does differ between small and major hospitals [16]. We also analyzed the data for every year in order to visualize time effects.

Because we expected ratings to be influenced quite a while before the intensified supervision, we categorized the intensified supervision period into 5 categories: the period before 1 year before intensified supervision, the 12 months before intensified supervision, during intensified supervision, the 12 months after intensified supervision, and the period after 1 year after intensified supervision. The period during intensified supervision varied according to the decision of the inspectorate to prolong intensified supervision (3-12 months; mean 7 months).

We performed a longitudinal logistic regression analysis (mixed model) to analyze whether periods of intensified supervision lead to lower patient ratings. To account for clustering of ratings within hospitals, “hospital” was included in the model as the random effect. The outcome variable was the dichotomized rating score.

Determinants in our analysis were the period of intensified supervision, hospital type, and the course of ratings over time (variable “time” in years). We included the factor time to analyze time trends that occur anyway, irrespective of intensified supervision.

### Ethical Approval

No ethical approval was needed because we used publicly available data and no persons were directly involved.

## Results

Table 1 lists the numbers of ratings for intensified supervision hospitals and control group hospitals per period and per hospital type. Table 2 presents the mean rating score and percentage of ratings lower than 6.5 per period for intensified supervision hospitals and control group hospitals. Table 3 presents the mean rating in time and the percentage of ratings lower that 6.5, for both groups.

**Table 1:** Numbers of rating scores per hospital type and period.

Hospitals and period		General acute care hospital		Teaching hospital		Academic hospital		Total	
		N hospitals	N ratings	N hospitals	N ratings	N hospitals	N ratings	N hospitals	N ratings
Control group hospitals		16	17,569	8	17,926	4	3967	28	39,462
Intensified supervision hospitals		4		2		1		7	
	Period before 1 year before intensified supervision		499		161		81		741
	1 year before intensified supervision		303		165		82		550
	During intensified supervision		285		106		53		444
	1 year after intensified supervision		272		313		94		679
	Period after 1 year after intensified supervision		604		1144		232		1980
Total		20	19,532	10	19,815	5	4509	35	43,856

**Table 2:** Mean rating score and percentage of ratings <6.5 per period for both intensified supervision and control group hospitals.

Hospitals and period		Mean rating score	Percentage of ratings <6.5
Control group hospitals		8.5	8.3%
Intensified supervision hospitals			
	Period before 1 year before intensified supervision	7.9	18.4%
	1 year before intensified supervision	8.1	16.5%
	During intensified supervision	8.2	14.9%
	1 year after intensified supervision	8.2	15.9%
	Period after 1 year after intensified supervision	8.5	10.6%

**Table 3:** Mean rating in time and percentage <6.5 for both intensified supervision and control group hospitals.

Year	Mean rating (%<6.5) intensified supervision hospitals	Mean rating (%<6.5) control group hospitals
2010	7.7 (19.1)	7.6 (19.9)
2011	7.8 (22.6)	8.2 (13.6)
2012	8.2 (15.6)	8.5 (8.6)
2013	8.5 (10.7)	8.7 (6.4)
2014	8.4 (12.6)	8.6 (7.9)
2015	8.4 (12.8)	8.7 (7.2)

Table 4 presents the results of the multilevel analysis. The period of supervision is associated with a low rating score for intensified supervision hospitals compared with control group hospitals: both 1 year before intensified supervision (odds ratio, OR, 1.67, 95% CI 1.06-2.63) and 1 year after intensified supervision (OR 1.79, 95% CI 1.14-2.81) differ significantly. For all periods the odds on a low rating score for intensified supervision hospitals are higher than for the control group hospitals, corrected for time. The proportion of low rating scores decreased over time since 2010.

**Table 4:** Effect of intensified supervision period on low patient ratings, adjusted for time and type of hospital.

Variables	Categories	Odds ratios	95% confidence interval	
			Lower	Upper
	Control group hospitals	1.0^a^		
Intensified supervision period	Period before 1 year before intensified supervision	1.29	0.825	2.028
	1 year before intensified supervision	1.67	1.059	2.634
	During intensified supervision	1.55	0.964	2.506
	1 year after intensified supervision	1.79	1.143	2.805
	Period after 1 year after intensified supervision	1.41	0.921	2.161
Time	2010	1.0^a^		
	2011	0.77	0.656	0.908
	2012	0.49	0.418	0.572
	2013	0.35	0.3	0.412
	2014	0.45	0.387	0.522
	2015	0.42	0.364	0.492
Hospital type	General acute	0.86	0.597	1.232
	Academic	1.1	0.659	1.836
	Teaching	1.0^a^		

^a^ Reference category.

## Discussion

On the basis of the results of this study, we can confirm the hypothesis that the average rating of intensified supervision hospitals before the intensified supervision started is lower compared with the control group. We found also that patient ratings were low not only before but also during and just after the intensified supervision: the scores are continuously relatively low. It seems that only during the period after a year after intensified supervision the ratings are comparable with the control group hospitals. This result might assure the inspectorate that intensified supervision does not influence the patient ratings that it uses for supervision significantly in the short term. The results do not indicate that the mean rating will increase during the intensified supervision, for example, because of the loyalty of patients.

Health care inspectorates might learn from these results because they indicate that the inspectorate identifies the same hospitals as “at risk” as the patients rate as underperformers. This can be seen as another indication of the opportunities for patients to identify patient safety problems [17,18]. Monthly monitoring of scores on PRSs by health care inspectorates or other quality monitoring organizations could be of additional value in identifying health care providers at risk. This is also in line with the results of several studies that show correlations between patient rating scores and quality indicators, although the correlations were mostly weak and sometimes inconsistent [6]. The potential contribution of patient rating scores to health care governance supports the initiatives of health care inspectorates already using these online scores in their daily supervision [8].

Although not part of the research question, we identified a trend in patient rating scores. Compared with the start in 2010, the mean overall ratings on PRS Zorgkaart Nederland have increased significantly in 2015. To the best of our knowledge, there has not been an analysis of the rating trend over several years. This might be due to relative low ratings of pioneers at the start of the PRS. Now that the PRS is used more, it might attract a broader public with more positive ratings in general. In total, 92% of the ratings in our dataset are positive (≥6.5), which is comparable with approximately 90% of ratings found in other research [19].

### Strengths and Limitations

An important strength of this study is the major database used with 43,856 online ratings of 35 hospitals, more than a third of all Dutch hospitals.

In addition, it takes into account the opportunities of using patient experiences in supervision of health care. Especially with the increasing use of rating sites in the near future when the generation socialized with social media (eg, Facebook and Twitter) reaches the age in which health questions and doctors become significant, these kinds of sources might become even more relevant for patients, physicians, and other stakeholders such as health care inspectorates [2,4,20]. An increasing number of patients are given a voice to their experiences in order to identify patient safety risks. Health care inspectorates all over the world might benefit from this and better involve citizens in health care governance.

A limitation of the study is that we only selected the hospitals with sufficient reviews in the control group. We cannot rule out that this was a selected group of hospitals. Furthermore, the number of hospitals with intensified supervision was low. There were only 7 hospitals confronted with intensified supervision. However, we analyzed the data on the level of patient ratings, which provided us with a dataset with enough power (n=43,856). The number of hospitals under supervision is only a small part of all providers in the Dutch health care with intensified supervision. In 2013 and 2014 it was concerned with 47 health care organizations of which 3 were hospitals. A second limitation is that at the moment the number of ratings of the other health care providers, mainly long-term elderly care, is too low to use for analysis. However, this could change rapidly. The branch organization of long-term elderly care announced in July 2015 the decision to cooperate with the Dutch Patient and Consumer Federation, the owner of Zorgkaart Nederland, in order to increase the number of reviews substantially by collecting ratings via interviews.

### Future Research

We expect that an increasing number of patients will share their experiences on PRSs and a growing number of patients will use those experiences in their judgment and choice of health care providers. This study suggests that all those experiences could be useful in estimating the quality of care because of the interesting association with the judgment of health care inspectors. However, it takes extensive research to understand this relationship better. Because of this association, this study might encourage health care inspectorates in experimenting with civilians as layman inspectors. It would also be interesting to know what underlying aspect of the IGZ's decision the negative patient reviews may be correlating with.

Exploring the reasons for low patient ratings in general will be necessary to give hospitals insight into how to improve their ratings. This could be investigated by closely studying the texts of the reviews, for example, by using Web-based text processing tools [21]. It might also be instructive for hospitals and IGZ to explore if any of the 6 aspects that go into the overall patient rating on Zorgkaart Nederland are more specifically correlated to intensified supervision rather than the overall score.

It might also be useful to explore the use of online patient ratings by health care parties other than inspectorates, for example, health insurance companies. They could start using rating scores to select preferred providers in their purchase of care.

Finally, research on the influence of supervisory activities should also be performed with other health care providers than hospitals, such as long-term care institutions, under the condition of sufficient number of reviews. Moreover, it would be relevant to repeat the study in other countries to investigate whether a different system of supervision or the presence of several PRSs influences the results.

### Conclusions

Hospitals that are confronted with intensified supervision by the health care inspectorate have lower ratings on PRSs. Health care inspectorates might learn from these results because they indicate that the inspectorate identifies the same hospitals as “at risk” as the patients rate as underperformers. More research with more ratings also in other parts of health care and other countries is needed to explore further the association between ratings on PRSs and the quality judgment of a health care inspectorate.

## Abbreviations

IGZ: Health Care Inspectorate (Inspectie voor de Gezondheidszorg)
OR: odds ratio
PRS: patient rating site
